# A Mathematical Theory of Phase-Consistent Information Bottleneck for Cross-Domain Generalization

**DOI:** 10.3390/e28070764

**Published:** 2026-07-03

**Authors:** Feng Liu, Zheng Wang

**Affiliations:** School of Information Engineering, Shandong Youth University of Political Science, Jinan 250103, China

**Keywords:** information bottleneck, dual-tree complex wavelet transform, domain generalization, disentangled representation, phase consistency, variational inference, medical image segmentation

## Abstract

We propose a mathematical framework for domain generalization in medical image segmentation built on dual-tree complex wavelet transform (DTCWT) and variational information theory. The core premise is that, under adequate spatial normalization and acquisition-style shifts, DTCWT phase components are more closely associated with anatomical structure, whereas amplitude components are more sensitive to domain-specific intensity and style variations. We formulate this as a local phase–magnitude complementarity premise and construct an information bottleneck that operates on structured subband representations. The framework provides several key theoretical results under explicit structural assumptions: an information bound showing when DTCWT amplitude subbands better isolate domain-related information than global Fourier representations; a variational information bottleneck encoder that compresses domain-specific amplitude information into low-dimensional latent codes; a triple constraint mechanism (domain supervision, KL compression, and orthogonality) that controls domain–task information leakage; and a predictive feature modulation scheme with O(1) spatial complexity. We further analyze test-time adaptation via calibrated uncertainty, deriving a sufficient condition under which a two-pass inference strategy reduces the expected generalization gap. Finally, we include illustrative public-dataset checks on FeTS 2022 and BraTS 2023 to test the central phase–amplitude premise and the feasibility of DTCWT-front-end segmentation. All theorems are stated with their assumptions and verifiable conditions, offering a physically motivated approach to domain generalization in medical imaging.

## 1. Introduction

Medical image segmentation models are commonly trained on data collected from a limited number of hospitals, scanners, and acquisition protocols. When such models are deployed at a new institution, the anatomical structures of interest may remain clinically comparable, but image contrast, bias fields, noise statistics, reconstruction artifacts, and intensity normalization can change substantially. This mismatch is a practical obstacle for brain tumor, organ, and lesion segmentation: a model can learn accurate boundaries on the source cohort yet fail on a target site because the same tissue class appears with different local intensity and texture characteristics.

The central question of this paper is therefore not only how to improve a network architecture, but how to describe domain shift in a representation where the problem becomes analyzable. We use the dual-tree complex wavelet transform (DTCWT) as such a representation. The working premise is that, after spatial normalization, local phase is more closely tied to anatomical structure, whereas local amplitude is more sensitive to acquisition style and scanner-dependent appearance. The rest of the paper develops a conditional information-theoretic framework around this premise, making explicit where standard tools are used and where new theoretical claims are introduced.

### 1.1. The Domain Generalization Problem

Let (X,Y) denote the input and label spaces. We consider *K* source domains $D_{S}=\left\{D_{1},\ldots ,D_{K}\right\}$, where each domain

(1) $$D_{k}={\left\{\left(x_{i}^{k},y_{i}^{k}\right)\right\}}_{i=1}^{N_{k}},\;x_{i}^{k}\in X,\;y_{i}^{k}\in Y$$

is drawn from an unknown joint distribution $P_{k}\left(x,y\right)$. The domain generalization objective is to learn a hypothesis h:X→Y that minimizes the risk on an unseen target domain $D_{T}∼P_{T}$:

(2) $$R_{T}\left(h\right)=E_{\left(x,y\right)∼P_{T}}\left[\ell \left(h\left(x\right),y\right)\right]$$

using only samples from $D_{S}$, without any access to target labels.

The following prior-work bound is used only as theoretical motivation. The classical Ben-David et al. generalization bound [1] provides the starting point:

(3) $$R_{T}\left(h\right)\leq R_{S}\left(h\right)+\frac{1}{2}d_{H\Delta H}\left(P_{S},P_{T}\right)+\lambda^{*}$$

where $d_{H\Delta H}$ is the HΔH-divergence between source and target distributions, and $\lambda^{*}$ is the combined optimal error.

### 1.2. The Frequency-Domain Structure of Domain Shift

A key structural observation, dating back to Oppenheim and Lim [2], is that in many signal processing contexts, the Fourier phase spectrum carries structural information while the magnitude spectrum carries intensity and style information. We extend this intuition to the medical imaging domain as a structural assumption whose validity depends on spatial normalization and on domain shifts that primarily affect acquisition appearance rather than gross anatomy.

Consider an invertible frequency-domain transform F:X→Z, where Z is a complex-valued space admitting polar decomposition $F\left(x\right)=A⊙e^{i\Phi }$ with amplitude A and phase Φ.

**Property 1**  (Conditional Frequency-Domain Domain Shift)**.** *For two domains $D_{k},D_{l}$ whose shift is dominated by acquisition appearance rather than gross anatomical change, the following approximate relationships are expected to hold under spatial normalization:*

(4) $$\left|F\left(x^{k}\right)\right|\left(u,v\right)\neq \left|F\left(x^{l}\right)\right|\left(u,v\right)\;(\mathrm{amplitude}\;\mathrm{varies}\;\mathrm{across}\;\mathrm{domains})$$

(5) $$∠F\left(x^{k}\right)\left(u,v\right)\approx ∠F\left(x^{l}\right)\left(u,v\right)\;(\mathrm{phase}\;\mathrm{is}\;\mathrm{approximately}\;\mathrm{invariant})$$

Equation (4) captures the observation that MRI contrast, gain, and intensity distributions can differ systematically across institutions. Equation (5) reflects the weaker and explicitly conditional premise that anatomical structures—organ boundaries, lesion contours, tissue interfaces—are more stable than acquisition appearance after spatial normalization.

### 1.3. Limitations of Global Fourier Approaches

Existing frequency-domain domain generalization methods [3,4] operate on the global Fourier transform $F_{\mathrm{FFT}}$. While computationally convenient, global FFT suffers from a fundamental tension:

**Proposition 1**  (Spatial Locality Failure of Global FFT)**.** *Let $x\in R^{H\times W}$ and let $\tilde{x}$ be a spatially localized modification of x on a compact region Ω⊂supp(x) with |Ω|≪HW. Under the global FFT amplitude modification $A_{\mathrm{FFT}}\mapsto {\tilde{A}}_{\mathrm{FFT}}$ performed on the entire frequency plane, the perturbation satisfies*

(6) $${∥\tilde{x}-x∥}_{F}^{2}={∥{\tilde{A}}_{\mathrm{FFT}}⊙e^{i\Phi_{\mathrm{FFT}}}-A_{\mathrm{FFT}}⊙e^{i\Phi_{\mathrm{FFT}}}∥}_{F}^{2}={∥{\tilde{A}}_{\mathrm{FFT}}-A_{\mathrm{FFT}}∥}_{F}^{2}$$

*where the Parseval equality implies that the spatial-domain perturbation energy is identical to the full-spectrum amplitude perturbation, regardless of spatial locality.*

This means: modifying any frequency bin affects every spatial pixel. Domain shifts in MRI are often spatially heterogeneous (e.g., frontal lobe brighter but occipital lobe normal in a particular scanner), and global FFT cannot isolate these local effects.

### 1.4. The Conditional Local Phase-Magnitude Complementarity Premise

The DTCWT [5] provides a solution through its multi-scale, multi-directional decomposition with approximate shift invariance. Its construction builds on complex and multidimensional dual-tree wavelets and the broader foundations of wavelet analysis [6,7,8,9]. We formalize the core physical insight:

**Property 2**  (Conditional Local Phase-Magnitude Complementarity in Medical Imaging)**.** *Let $x^{k},x^{l}$ be MRI images of the same anatomical structure acquired at different institutions. When the dominant cross-domain differences are intensity, contrast, bias-field, or reconstruction effects rather than anatomical deformation, the DTCWT W at scale j and orientation θ is assumed to satisfy:*

(7) $$\begin{array}{cc} ∠W{\left(x^{k}\right)}_{j,\theta } & \approx ∠W{\left(x^{l}\right)}_{j,\theta } \end{array}$$

(8) $$\begin{array}{cc} ∥W{\left(x^{k}\right)}_{j,\theta }∥ & =T_{k\to l}\left(∥W{\left(x^{l}\right)}_{j,\theta }∥\right) \end{array}$$

*where $T_{k\to l}$ is a spatially local, generally nonlinear energy redistribution operator acting on the amplitude manifold.*

Property 2 is the mathematical cornerstone of our framework. Under this premise, domain shift is structured in the DTCWT domain: phase is treated as a relatively anatomy-associated component, whereas amplitude is treated as an acquisition-sensitive and spatially localized component. It should be read as a conditional structural premise, not as an unconditional claim about all medical images. It is expected to hold best after spatial registration or resampling, and may fail under severe misregistration, major anatomical deformation, missing modalities, or cross-modality shifts where the imaging physics changes the structural signal itself.

### 1.5. Scope, Evidence, and Falsifiability of the Premise

Because subsequent results depend on Property 2, we explicitly separate three levels of claim. First, the premise is physically motivated: MRI site shifts often arise from acquisition-dependent contrast, gain, bias-field, and reconstruction differences, whereas anatomical boundaries are expected to be more stable after spatial normalization. Second, the premise is representation-specific: it is not a statement about raw pixels or global Fourier coefficients, but about local complex-wavelet coefficients whose phase and amplitude are spatially indexed. Third, the premise is empirically falsifiable: if DTCWT phase statistics predict acquisition site as strongly as, or more strongly than, amplitude statistics, or if DTCWT front ends fail to support non-trivial segmentation on public data, then the proposed decomposition would not be a useful basis for the theory.

The manuscript therefore treats the theorems below as conditional results. The empirical checks in Section 11 are not intended to prove a universal physical law; they test whether the premise is plausible in representative multi-site brain-tumor MRI settings. This distinction is important for interpretation: the theory provides sufficient conditions and failure-mode analysis, while the experiments assess whether the assumed conditions are reasonable enough to motivate further methodological development.

### 1.6. Contributions

This work makes several theoretical contributions. First, we formalize a conditional phase–magnitude domain structure under DTCWT and establish a bound on information loss introduced by axial-plane approximation. Second, we propose a variational information bottleneck tailored to structured DTCWT subbands and analyze how subband representations reduce the effective dimensionality of information compression compared to raw spatial inputs. Third, we derive a triple-constraint analysis for disentanglement, showing that domain supervision, KL compression, and orthogonality address distinct failure modes of task/domain separation. Fourth, we design a predictive modulation strategy that preserves information bottleneck semantics while lowering spatial parameter complexity to O(1) with respect to the amplitude volume size. Fifth, we present a theoretical analysis of uncertainty-guided test-time adaptation, deriving a sufficient condition for generalization gap reduction by a two-pass inference procedure. Finally, we add illustrative public-dataset validation to test the phase–amplitude premise and demonstrate DTCWT-front-end segmentation feasibility without reframing the paper as a full benchmark study.

## 2. Related Work

### 2.1. Frequency-Domain Domain Generalization

Frequency-domain methods have been used to reduce domain shift by manipulating Fourier amplitude while preserving Fourier phase. Fourier Domain Adaptation (FDA) replaces low-frequency amplitude components between source and target images for semantic segmentation [3], and later Fourier-based domain generalization work extends this idea without requiring target labels [4,10]. These methods motivate the use of phase–amplitude structure, but their global Fourier basis does not localize domain shifts in space. Our framework differs by using DTCWT subbands, which provide multiscale and multidirectional localization.

### 2.2. Information Bottleneck and Information-Theoretic DG

The information bottleneck principle [11] and its variational implementation [12] provide the compression language used in this paper. Related studies have examined information-theoretic representations, noisy compression, and the limits of information-bottleneck explanations in deep learning [13,14,15]. Recent information-theoretic domain generalization methods are directly relevant. Invariant information bottleneck (IIB) formulates domain generalization through invariant causal prediction and mutual-information regularization [16]. INSURE further uses information-theoretic disentanglement and purification to separate class-relevant and auxiliary domain information [17]. Our contribution is complementary: instead of applying the bottleneck directly to generic deep features, we impose it on physically structured wavelet amplitude subbands.

### 2.3. Invariant, Domain-Specific, and Causal Representations

Classical domain generalization methods seek invariant feature representations [18,19,20,21,22]. However, recent work shows that domain-specific information can also be useful when it is separated from invariant task information. The mDSDI framework explicitly learns both domain-invariant and domain-specific components to improve generalization [23]. This view is close to our use of $Z_{\mathrm{task}}$ and $Z_{\mathrm{domain}}$: domain information is not discarded, but compressed and isolated so that it can support controlled modulation rather than leak into task prediction. The distinction between useful factorization and unsupported statistical independence is also consistent with the broader disentanglement literature, which shows both the utility and the identifiability limitations of unsupervised latent-factor separation [24,25,26].

### 2.4. Foundation Models and Test-Time Adaptation

Segmentation foundation models such as SAM [27] and MedSAM [28] improve cross-task transfer by large-scale pretraining and promptability. They address a different axis of generalization from the present work. Our analysis focuses on site and acquisition shifts in a fixed medical segmentation setting, and studies how a physically motivated frequency representation can make such shifts analyzable. Similarly, uncertainty-guided test-time adaptation methods use predictive uncertainty as a deployment-time signal; Section 10 provides a conditional analysis of this idea in the DTCWT amplitude domain.

### 2.5. Foundational Tools Versus Proposed Contributions

Several equations used below are standard and are included to make the paper self-contained. Table 1 separates established tools from the contributions developed in this work.

To further clarify the methodological position of this paper, Table 2 compares the proposed framework with representative domain generalization and segmentation paradigms. The comparison is not intended to claim state-of-the-art empirical performance; rather, it identifies which mathematical object each framework controls and where the present analysis adds a distinct signal-structured bottleneck.

Table 3 makes the boundary between inherited tools and original theoretical claims explicit. Standard equations are used only as preliminaries or implementation losses; the original contribution is the conditional placement of these tools in a DTCWT-structured phase–amplitude representation.

## 3. Preliminaries

### 3.1. Notation

Let [n]={1,…,n}. For vectors $a,b\in R^{d}$, $\langle a,b\rangle =a^{⊤}b$ denotes the inner product, and ${∥a∥}_{2}=\sqrt{\langle a,a\rangle }$. The Frobenius norm of a matrix M is ${∥M∥}_{F}=\sqrt{{\langle M,M\rangle }_{F}}$ where ${\langle A,B\rangle }_{F}=\mathrm{tr}\left(A^{⊤}B\right)$. For a complex number z=a+ib, $\left|z\right|=\sqrt{a^{2}+b^{2}}$ and ∠z=atan2(b,a).

We use N(μ,Σ) for the multivariate normal distribution and KL(·∥·) for the Kullback–Leibler divergence. The mutual information between random variables *X* and *Y* is denoted I(X;Y), following standard information-theoretic notation [29].

### 3.2. Information Bottleneck Foundations

The equations in this subsection are prior-work foundations included for self-containment. The classical information bottleneck (IB) principle [11] seeks a compressed representation *Z* of input *X* that preserves maximal information about a target *Y*:

(9) $$\min_{p(z|x)}I\left(X;Z\right)-\beta I\left(Z;Y\right)$$

where β>0 trades off compression against prediction.

The variational information bottleneck (VIB) [12] provides a tractable variational approximation:

(10) $$L_{\mathrm{VIB}}=E_{x,y}\left[E_{z∼q_{\phi }\left(z|x\right)}\left[-\log p_{\theta }\left(y|z\right)\right]\right]+\beta \cdot \mathrm{KL}\left(q_{\phi }\left(z|x\right)∥p\left(z\right)\right)$$

where $q_{\phi }\left(z|x\right)=N\left(\mu_{\phi }\left(x\right),\Sigma_{\phi }\left(x\right)\right)$ is a learned stochastic encoder and p(z)=N(0,I) is a fixed prior.

### 3.3. Domain Generalization Bounds

A central result in domain generalization theory is the bound:

(11) $$\forall h\in H:R_{T}\left(h\right)\leq \frac{1}{K}\sum_{k=1}^{K}R_{k}\left(h\right)+d\left({\left\{P_{k}\right\}}_{k=1}^{K},P_{T}\right)+\lambda$$

where d(·,·) measures the divergence between the mixture of source distributions and the target distribution [30].

## 4. DTCWT: Structured Phase-Amplitude Representation

### 4.1. Axial-Plane Complex Wavelet Decomposition

For a 3D medical image $x\in R^{4\times D\times H\times W}$ (four-channel MRI with depth *D*, height *H*, width *W*), we define the axial-plane DTCWT slice-wise.

**Definition 1**  (Axial-Plane 2D DTCWT)**.** *For axial slice z∈[D], let $x_{z}\in R^{4\times H\times W}$. The DTCWT uses a pair of Q-shift dual-tree filter banks $\left\{h_{0}^{\left(a\right)},h_{1}^{\left(a\right)}\right\}$ and $\left\{h_{0}^{\left(b\right)},h_{1}^{\left(b\right)}\right\}$ that form the real and imaginary parts of an approximately analytic complex wavelet. At scale j∈[J] and orientation $\theta \in \{\pm 15^{\circ },\pm 45^{\circ },\pm 75^{\circ }\}$, the complex subband coefficient is*

(12) $$W_{j,\theta }\left(x_{z}\right)=W_{j,\theta }^{\left(a\right)}\left(x_{z}\right)+i\cdot W_{j,\theta }^{\left(b\right)}\left(x_{z}\right)\in C^{4\times D_{j}\times H_{j}}$$

*where $D_{j}=\left\lfloor D/2^{j}\right\rfloor$, $H_{j}=\left\lfloor H/2^{j}\right\rfloor$ are the downsampled spatial dimensions.*

**Definition 2**  (Subband Amplitude and Phase)**.** *For each complex subband $W_{j,\theta }$, the amplitude and phase are*

(13) $$A_{j,\theta }=\left|W_{j,\theta }\right|\in R_{+}^{4\times D_{j}\times H_{j}},\;\Phi_{j,\theta }=∠W_{j,\theta }\in {\left[-\pi ,\pi \right)}^{4\times D_{j}\times H_{j}}$$

Stacking over all slices along the *z*-axis yields 3D amplitude and phase volumes:

(14) $$A={\left\{A_{j,\theta }\right\}}_{j,\theta }\in R_{+}^{C_{\mathrm{amp}}\times D_{J}\times H_{J}\times D},\;\Phi ={\left\{\Phi_{j,\theta }\right\}}_{j,\theta }\in {\left[-\pi ,\pi \right)}^{C_{\mathrm{amp}}\times D_{J}\times H_{J}\times D}$$

where $C_{\mathrm{amp}}=4\times J\times 6$ is the total number of amplitude channels across all scales and orientations.

**Remark 1**  (Axial Approximation)**.** *The axial-plane 2D DTCWT processes each slice independently, without explicit through-plane directional selectivity. This is a clinically motivated approximation: MRI is acquired as an axial slice-stack where through-plane resolution is typically coarser than in-plane resolution [31].*

**Proposition 2**  (Information Loss of Axial Approximation)**.** *Let $W_{3D}$ denote a true 3D DTCWT with 28 directional subbands and $W_{2D}^{z}$ the axial-plane 2D approximation. Under the assumption that through-plane spatial frequencies are bandlimited by the acquisition point-spread function with cutoff $\omega_{z}^{\max}$, the mutual information gap satisfies*

(15) $$I\left(x;W_{3D}\left(x\right)\right)-I\left(x;W_{2D}^{z}\left(x\right)\right)\leq H\left(\frac{\omega_{z}^{\max}}{\omega_{xy}^{\max}}\right)\cdot \log\left(28/6\right)$$

*where H(p)=−plogp−(1−p)log(1−p) is the binary entropy function and $\omega_{xy}^{\max}$ is the in-plane frequency cutoff.*

**Proof.**  The gap arises from the 28 vs. 6 directional subbands in the through-plane direction. Under bandlimitedness, the through-plane frequency content contributes proportionally $\omega_{z}^{\max}/\omega_{xy}^{\max}$ to the total signal entropy. The subband count ratio 28/6 multiplies the directional information capacity. Applying the data processing inequality and the entropy bound yields the result.    □

### 4.2. Phase-Amplitude Coupling and the Need for $L_{\mathrm{phase}}$

An important technical nuance: DTCWT amplitude and phase are not perfectly independent.

**Lemma 1**  (Amplitude-Phase Coupling)**.** *Let $\tilde{A}\neq A$ be a modified amplitude spectrum. The phase of the reconstructed and re-transformed signal satisfies*

(16) $$∠W{\left(W^{-1}(\tilde{A}⊙e^{i\Phi })\right)}_{j,\theta }=\Phi_{j,\theta }+\epsilon_{j,\theta }\left(\tilde{A},A\right)$$

*where $\epsilon_{j,\theta }\in {\left[-\delta ,\delta \right]}^{4\times D_{j}\times H_{j}}$ is a bounded phase drift with $\delta =O(∥\tilde{A}-A{∥}_{F}/∥A{∥}_{F})$.*

**Proof.**  The DTCWT inverse $W^{-1}$ involves linear combinations of subband coefficients through synthesis filters. When $\tilde{A}\neq A$, the synthesis integration over orientations mixes phase contributions. However, the Q-shift design ensures near-orthogonality between orientations [5], bounding the cross-orientation leakage. Taylor expansion of the phase perturbation yields $\epsilon_{j,\theta }=\nabla_{A}\Phi_{j,\theta }\cdot \left(\tilde{A}-A\right)+O(∥\tilde{A}-A{∥}^{2})$, where $∥\nabla_{A}\Phi ∥\leq \kappa /∥A∥$ for some constant κ determined by the filter bank design.    □

### 4.3. Domain Information Concentration in Amplitude Subbands

We now establish the information-theoretic justification for operating the VIB on amplitude subbands.

**Theorem 1**  (Domain Information in Amplitude Subbands)**.** *Let D∈[K] denote the domain identity random variable and A the DTCWT amplitude representation. Under Property 2 and mild regularity conditions on $T_{k\to l}$, the domain-relevant mutual information satisfies*

(17) $$I\left(A;D\right)\geq I\left(F_{\mathrm{FFT}}\left(x\right);D\right)-\Delta_{\mathrm{FFT}}$$

*where $\Delta_{\mathrm{FFT}}=I\left(x;D|F_{\mathrm{FFT}}\left(x\right)\right)$ is the domain information lost by the global FFT representation.*

**Proof Sketch. **  The global FFT discards spatial locality: $|F_{\mathrm{FFT}}\left(x\right)|\left(u,v\right)$ aggregates energy over the entire spatial support. For a spatially localized domain shift (e.g., scanner-dependent bias field in the frontal region), the FFT amplitude at frequency (u,v) receives contributions from both the shifted and unshifted regions, diluting the domain signal. The DTCWT, by contrast, provides spatial localization through its compact-support basis functions. Specifically, the effective spatial support of $W_{j,\theta }$ at scale *j* is $O(2^{j}\times 2^{j})$—much smaller than the full image. By the data processing inequality applied to the spatially localized subbands:

(18) $$I\left(A;D\right)=I\left({\left\{A_{j,\theta }\right\}}_{j,\theta };D\right)\geq \max_{j,\theta }I\left(A_{j,\theta };D\right)=I\left(D;D\right)-\Delta_{\mathrm{FFT}}$$

where the inequality follows from the fact that each subband independently captures a portion of the domain information.    □

## 5. Variational Information Bottleneck on Structured Subbands

### 5.1. Encoder Architecture

We now construct the VIB encoder operating on DTCWT amplitude subbands.

**Definition 3**  (Amplitude VIB Encoder)**.** *Given amplitude volume $A\in R_{+}^{C_{\mathrm{amp}}\times D_{J}\times H_{J}\times D}$, the encoder $q_{\phi }\left(Z_{\mathrm{domain}},Z_{\mathrm{task}}|A\right)$ is defined through*

(19) $$\begin{array}{cc} h & ={\mathrm{MLP}}_{\mathrm{enc}}\left(\mathrm{GAP}(A)\right)\in R^{d_{h}} \end{array}$$

(20) $$\begin{array}{cc} \mu ,\log\sigma & ={\mathrm{Linear}}_{\mu }\left(h\right),{\mathrm{Linear}}_{\sigma }\left(h\right)\in R^{d_{z}} \end{array}$$

(21) $$\begin{array}{cc} Z_{\mathrm{domain}} & ∼N(\mu ,\mathrm{diag}\left(\sigma^{2}\right)) \end{array}$$

(22) $$\begin{array}{cc} Z_{\mathrm{task}} & ={\mathrm{Linear}}_{\mathrm{task}}\left(h\right)\in R^{d_{z}} \end{array}$$

*where GAP denotes global average pooling over the spatial dimensions and $d_{z}$ is the latent dimension.*

### 5.2. Variational Bound on Domain Information

The following analysis combines the VIB variational bound with standard information-theoretic and PAC-Bayesian generalization arguments [32,33].

**Theorem 2**  (VIB Upper Bound on Domain-Attribute Mutual Information)**.** *Let $q_{\phi }\left(Z_{\mathrm{domain}}|A\right)$ be the encoder defined above with prior $p\left(Z_{\mathrm{domain}}\right)=N\left(0,I\right)$. For any test function family G, the mutual information between the domain representation and amplitude satisfies*

(23) $$I\left(Z_{\mathrm{domain}};A\right)\leq \frac{1}{N}\sum_{i=1}^{N}\mathrm{KL}\left(q_{\phi }\left(Z_{\mathrm{domain}}|A_{i}\right)∥p\left(Z_{\mathrm{domain}}\right)\right)+\log\frac{\left|G\right|}{\delta }$$

*with a probability of at least 1−δ over the training sample of size N, where |G| is a complexity measure of the encoder class.*

**Proof.**  The mutual information admits the variational upper bound

(24) $$\begin{array}{cc} I(Z_{\mathrm{domain}};A) & =E_{p(A,Z_{\mathrm{domain}})}\left[\log\frac{q_{\phi }\left(Z_{\mathrm{domain}}|A\right)}{p(Z_{\mathrm{domain}})}\right]-\mathrm{KL}\left(p\left(Z_{\mathrm{domain}}\right)∥p\left(Z_{\mathrm{domain}}\right)\right)\\ \; & \leq E_{p(A)}\left[\mathrm{KL}(q_{\phi }\left(Z_{\mathrm{domain}}|A\right)∥p\left(Z_{\mathrm{domain}}\right))\right] \end{array}$$

The empirical estimation introduces a finite-sample penalty. By McAllester’s PAC-Bayesian bound [32], applied to the KL estimator with function class G and confidence δ, we obtain the stated result.    □

Theorem 2 provides the theoretical justification for using the KL divergence as a computable upper bound on domain-relevant information in the latent representation.

### 5.3. Information Sufficiency for Segmentation

We must also ensure that compressing domain information does not destroy task-relevant information.

**Theorem 3**  (Conditional Task Information Preservation)**.** *Let $Z_{\mathrm{task}}$ be the deterministic task representation, $Z_{\mathrm{domain}}$ the domain representation, and y the segmentation label. Assume that the combined representation $\left(Z_{\mathrm{task}},Z_{\mathrm{domain}}\right)$ is $\epsilon_{s}$-sufficient for the amplitude representation in the sense that*

(25) $$I\left(A;y|Z_{\mathrm{task}},Z_{\mathrm{domain}}\right)\leq \epsilon_{s},$$

*and that residual domain-to-label leakage after conditioning on $Z_{\mathrm{task}}$ is bounded by*

(26) $$I(Z_{\mathrm{domain}};y|Z_{\mathrm{task}})\leq \eta .$$

*Then the task representation preserves segmentation-relevant information up to the sufficiency and leakage errors:*

(27) $$I\left(Z_{\mathrm{task}};y\right)\geq I\left(A;y\right)-\epsilon_{s}-\eta .$$

**Proof.**  Because $\left(Z_{\mathrm{task}},Z_{\mathrm{domain}}\right)$ is generated from A, the chain rule gives

(28) $$I\left(A;y\right)=I\left(Z_{\mathrm{domain}},Z_{\mathrm{task}};y\right)+I\left(A;y|Z_{\mathrm{domain}},Z_{\mathrm{task}}\right)$$

and

(29) $$I\left(Z_{\mathrm{domain}},Z_{\mathrm{task}};y\right)=I\left(Z_{\mathrm{task}};y\right)+I\left(Z_{\mathrm{domain}};y|Z_{\mathrm{task}}\right).$$

Substituting the second identity into the first and rearranging yields

(30) $$I\left(Z_{\mathrm{task}};y\right)=I\left(A;y\right)-I\left(A;y|Z_{\mathrm{task}},Z_{\mathrm{domain}}\right)-I\left(Z_{\mathrm{domain}};y|Z_{\mathrm{task}}\right).$$

Applying the assumed bounds $I\left(A;y|Z_{\mathrm{task}},Z_{\mathrm{domain}}\right)\leq \epsilon_{s}$ and $I(Z_{\mathrm{domain}};y|Z_{\mathrm{task}})\leq \eta$ gives the result. This theorem does not require orthogonality to imply statistical independence; orthogonality is used later only as a tractable finite-batch regularizer for reducing linear leakage.    □

## 6. Triple-Constraint Disentanglement Analysis

### 6.1. Domain Supervision

**Definition 4**  (Domain Classification Loss)**.** *This is the standard cross-entropy loss for supervised domain classification, used here as an identifiability signal for $Z_{\mathrm{domain}}$ rather than as a new loss formulation. Given training domains indexed by {1,…,K}, the domain supervision loss is*

(31) $$L_{\mathrm{dom}}=-\frac{1}{B}\sum_{b=1}^{B}\sum_{k=1}^{K}1_{\left[\mathrm{site}(b)=k\right]}\log{\hat{y}}_{b,k}$$

*where ${\hat{y}}_{b}=\mathrm{softmax}\left({\mathrm{MLP}}_{\mathrm{dom}}\left(Z_{\mathrm{domain}}^{\left(b\right)}\right)\right)\in \Delta^{K-1}$.*

**Proposition 3**  (Domain Supervision Identifiability)**.** *If $E[L_{\mathrm{dom}}]\to 0$, then as B→∞, the posterior $P(D=k|Z_{\mathrm{domain}})$ converges to a point mass on the true domain index.*

However, domain supervision alone is *insufficient* for disentanglement.

**Proposition 4**  (Failure Mode of Pure Domain Supervision)**.** *Let $L_{\mathrm{dom}}$ be the only domain-related loss term. Then there exist encoder configurations where*

(32) $$I\left(Z_{\mathrm{domain}};y\right)>0\;\mathrm{and}\;I\left(Z_{\mathrm{domain}};A\right)\to \infty$$

*i.e., $Z_{\mathrm{domain}}$ captures both domain and task information without bound.*

**Proof.**  Construct a pathological encoder that encodes the entire input into $Z_{\mathrm{domain}}$ with high precision: $Z_{\mathrm{domain}}=\mathrm{MLP}\left(A\right)$ with $\dim\left(Z_{\mathrm{domain}}\right)\gg \dim\left(A\right)$. Then ${\hat{y}}_{b}$ achieves perfect domain classification while $Z_{\mathrm{domain}}$ retains the full input information, including y. The cross-entropy loss provides no penalty for this information leakage.    □

### 6.2. KL Compression

**Definition 5**  (KL Compression Loss)**.** *This is the standard closed-form Gaussian KL term used in variational bottleneck models. The KL divergence between the domain posterior and the standard normal prior is*

(33) $$L_{\mathrm{KL}}=\mathrm{KL}\left(q_{\phi }\left(Z_{\mathrm{domain}}|A\right)∥N\left(0,I\right)\right)=\frac{1}{2}\sum_{j=1}^{d_{z}}\left(\mu_{j}^{2}+\sigma_{j}^{2}-\log\sigma_{j}^{2}-1\right)$$

**Proposition 5**  (KL Prevents Information Overload)**.** *Under the KL constraint, $E[L_{\mathrm{KL}}]\leq C$ implies $I(Z_{\mathrm{domain}};A)\leq C+\log N$ (by Theorem 2). This prevents the pathological encoder of Proposition 4.*

The $L_{\mathrm{dom}}+L_{\mathrm{KL}}$ pair forms a push–pull equilibrium:

- $L_{\mathrm{dom}}$ pulls $Z_{\mathrm{domain}}$ to contain domain-discriminative information;
- $L_{\mathrm{KL}}$ pushes $Z_{\mathrm{domain}}$ toward the uninformative prior, limiting total information capacity.

**Theorem 4**  (Idealized Push–Pull Equilibrium)**.** *Let $L_{\text{push-pull}}=L_{\mathrm{dom}}+\beta L_{\mathrm{KL}}$. In the idealized constrained information-bottleneck problem, for each attainable capacity level $C(\beta^{*})$, there exists a Lagrange multiplier $\beta^{*}$ such that any optimal encoder $q_{\phi }^{*}$ satisfies*

(34) $$I\left(Z_{\mathrm{domain}};D\right)=\max_{q:I\left(Z_{\mathrm{domain}};A\right)\leq C\left(\beta^{*}\right)}I\left(Z_{\mathrm{domain}};D\right)$$

*where C(β)→0 as β→∞ and C(β)→∞ as β→0.*

**Proof.**  This follows from the constrained optimization perspective of the information bottleneck under the standard regularity conditions for Lagrangian relaxation. The Lagrangian formulation $\min_{q}I\left(Z_{\mathrm{domain}};A\right)-\beta I\left(Z_{\mathrm{domain}};D\right)$ is equivalent to $\max_{q}I\left(Z_{\mathrm{domain}};D\right)$ subject to $I\left(Z_{\mathrm{domain}};A\right)\leq C\left(\beta \right)$. $L_{\mathrm{dom}}$ approximates $-I(Z_{\mathrm{domain}};D)$ and $L_{\mathrm{KL}}$ bounds $I(Z_{\mathrm{domain}};A)$ (Theorem 2). For neural-network implementations this result should be interpreted as an objective-level rationale rather than a guarantee of global optimization uniqueness.    □

However, the push–pull pair still does not guarantee separation between task and domain information within $Z_{\mathrm{domain}}$.

**Proposition 6**  (Failure Mode of Push–Pull Without Orthogonality)**.** *Even with optimal $\beta^{*}$, there exist encoder configurations where*

(35) $$\langle Z_{\mathrm{task}},Z_{\mathrm{domain}}\rangle \neq 0$$

*i.e., the task and domain subspaces are not orthogonal, allowing task information to leak into the domain code.*

### 6.3. Orthogonality Constraint

**Definition 6**  (Orthogonality Loss)**.** *The orthogonality constraint measures the cosine similarity between task and domain representations:*

(36) $$L_{\mathrm{orth}}=\frac{∥Z_{\mathrm{task}}^{⊤}Z_{\mathrm{domain}}{∥}_{F}^{2}}{∥Z_{\mathrm{task}}{∥}_{F}^{2}\cdot {∥Z_{\mathrm{domain}}∥}_{F}^{2}}$$

*where $Z_{\mathrm{task}},Z_{\mathrm{domain}}\in R^{B\times d_{z}}$ are batch matrices of task and domain latent codes.*

**Remark 2**  (Normalization)**.** *Equation (36) uses $L_{2}$-normalized cosine similarity ∈[0,1], decoupling the constraint from batch size. The Frobenius norm $∥Z_{\mathrm{task}}^{⊤}Z_{\mathrm{domain}}{∥}_{F}^{2}$ without normalization would scale linearly with B, introducing unwanted batch-size dependence.*

**Theorem 5**  (Orthogonality Controls Linear Cross-Subspace Leakage)**.** *Let $\tilde{Z_{\mathrm{task}}}$ and $\tilde{Z_{\mathrm{domain}}}$ denote batch-centered task and domain latent matrices. If $L_{\mathrm{orth}}=0$, then the empirical cross-covariance between the two latent subspaces vanishes:*

(37) $$\hat{\mathrm{Cov}}\left(Z_{\mathrm{task}},Z_{\mathrm{domain}}\right)=\frac{1}{B-1}{\tilde{Z_{\mathrm{task}}}}^{⊤}\tilde{Z_{\mathrm{domain}}}=0.$$

*Consequently, the domain code and task code have no linear cross-subspace leakage within the batch. If, in addition, the latent variables are jointly Gaussian and $Z_{\mathrm{domain}}$ is a minimal sufficient statistic for domain identity under the IB objective, this zero cross-covariance implies conditional information separation, $I(Z_{\mathrm{task}};D|y)=0$.*

**Proof.**  By definition, $L_{\mathrm{orth}}=0$ implies $Z_{\mathrm{task}}^{⊤}Z_{\mathrm{domain}}=0_{d_{z}\times d_{z}}$ after normalization. For centered batches this is exactly the empirical cross-covariance up to the factor ${\left(B-1\right)}^{-1}$. Thus no linear predictor can exploit a component of $Z_{\mathrm{task}}$ that is aligned with the domain subspace represented by $Z_{\mathrm{domain}}$. This statement does not, by itself, imply statistical independence. The mutual-information statement follows only under the additional linear-Gaussian condition, where zero covariance is equivalent to independence, together with the minimal sufficiency assumption that all domain-discriminative information available to the encoder is allocated to $Z_{\mathrm{domain}}$.    □

### 6.4. Non-Redundancy of the Triple Guarantee

**Theorem 6**  (Conditional Non-Redundancy of the Triple Constraint)**.** *The three constraints $\left(L_{\mathrm{dom}},L_{\mathrm{KL}},L_{\mathrm{orth}}\right)$ address distinct and non-redundant failure modes of the conditional disentanglement objective:*

(38) $$I\left(Z_{\mathrm{domain}};D\right)>0\;\wedge \;I\left(Z_{\mathrm{domain}};A\right)\leq \epsilon \;\wedge \;\hat{\mathrm{Cov}}\left(Z_{\mathrm{task}},Z_{\mathrm{domain}}\right)=0.$$

*Under the additional linear-Gaussian and minimal sufficiency assumptions of Theorem 5, the third condition yields conditional information separation.*

**Proof Structure.**  We establish the result by identifying the failure mode removed by each constraint:

(i) Joint effect: $L_{\mathrm{dom}}+L_{\mathrm{KL}}+L_{\mathrm{orth}}\to 0$ implies domain identifiability, bounded latent capacity, and vanishing linear cross-subspace leakage.
(ii) Necessity of $L_{\mathrm{dom}}$: Without $L_{\mathrm{dom}}$, $Z_{\mathrm{domain}}$ may collapse to prior N(0,I), losing all domain information.
(iii) Necessity of $L_{\mathrm{KL}}$: Without $L_{\mathrm{KL}}$, $Z_{\mathrm{domain}}$ may encode the full input (Proposition 4).
(iv) Necessity of $L_{\mathrm{orth}}$: Without $L_{\mathrm{orth}}$, domain and task subspaces may overlap (Proposition 6).

Each counterexample is constructed explicitly: the proofs of Propositions 4 and 6 serve as the necessity arguments.    □

Theorem 6 is the central theoretical result of this paper. It establishes that each constraint addresses a distinct failure mode. The result is conditional: orthogonality controls linear leakage in general and implies statistical separation only under the additional distributional assumptions stated in Theorem 5.

### 6.5. Annealing Schedule for β

**Definition 7**  (KL Annealing)**.** *The coefficient β(t) is not a fixed hyperparameter but an annealing schedule:*

(39) $$\beta \left(t\right)=\min\left(1,\frac{t}{T_{\mathrm{anneal}}}\right),\;t\in \left\{0,\ldots ,T-1\right\}$$

This prevents posterior collapse in early training: at t=0, β=0 allows the encoder to freely learn informative representations; as β→1, the KL constraint gradually tightens.

## 7. Predictive Feature Modulation: O(1) Complexity

### 7.1. Generative Decoder Infeasibility

In the classical VIB framework, a generative decoder $p_{\theta }\left(x|Z\right)$ reconstructs the input to verify information retention. For our setting:

**Proposition 7**  (Generative Decoder Complexity)**.** *A generative decoder mapping $Z_{\mathrm{domain}}\to A$ requires*

(40) $$\mathrm{params}\left(\mathrm{Decoder}\right)=\Omega \left(\prod_{s=1}^{3}\dim{\left(A\right)}_{s}\right)=\Omega \left(C_{\mathrm{amp}}\times D_{J}\times H_{J}\times D\right)$$

*For a $240^{3}$ input with J=2 DTCWT levels, this implies $\dim\left(A\right)\approx 4\times 2\times 6\times 60\times 60\times 240\approx 4.1\times 10^{7}$ dimensions, requiring a decoder with $∼10^{9}$ parameters.*

### 7.2. FiLM as Information-Consistent Alternative

**Definition 8**  (Predictive FiLM Modulation)**.** *The affine modulation form follows the standard FiLM operator; the proposed component is its use as a predictive, bottleneck-conditioned alternative to reconstructing the full DTCWT amplitude volume. Given bottleneck features $F\in R^{C\times D_{b}\times H_{b}\times W_{b}}$, the modulated features are*

(41) $$F_{\mathrm{mod}}=\gamma \left(Z_{\mathrm{task}},Z_{\mathrm{domain}}\right)⊙F+\beta \left(Z_{\mathrm{task}},Z_{\mathrm{domain}}\right)$$

*where $\gamma ,\beta \in R^{C}$ are channel-wise scale and shift parameters generated by a lightweight hypernetwork ${\mathrm{MLP}}_{\mathrm{FiLM}}:R^{2d_{z}}\to R^{2C}$.*

**Theorem 7**  (FiLM Preserves IB Semantics)**.** *The FiLM-based conditional distribution $p(F_{\mathrm{mod}}|Z_{\mathrm{task}},Z_{\mathrm{domain}})$ preserves the information bottleneck constraints:*

(42) $$\begin{array}{cc} I(Z_{\mathrm{domain}};F_{\mathrm{mod}}) & \leq I\left(Z_{\mathrm{domain}};A\right)\leq C\left(\beta \right) \end{array}$$

(43) $$\begin{array}{cc} I(Z_{\mathrm{task}};F_{\mathrm{mod}}) & \geq I(Z_{\mathrm{task}};y)-\epsilon \end{array}$$

*where C(β) is the KL-imposed capacity bound and ϵ accounts for the FiLM information loss (bounded by the network capacity).*

**Proof.**  Equation (42) follows from the data processing inequality: $Z_{\mathrm{domain}}\to A\to F_{\mathrm{mod}}$ forms a Markov chain. Equation (43): $Z_{\mathrm{task}}\to F_{\mathrm{mod}}\to y$ is the inference path. The FiLM transformation is Lipschitz-continuous (bounded weights), ensuring $I\left(Z_{\mathrm{task}};F_{\mathrm{mod}}\right)\geq I\left(Z_{\mathrm{task}};y\right)-\mathrm{Lip}\left(\mathrm{FiLM}\right)\cdot \epsilon$.    □

**Theorem 8**  (Complexity Reduction)**.** *Predictive FiLM modulation achieves*

(44) $$\mathrm{params}\left(\mathrm{FiLM}\right)=O\left(d_{z}\cdot C\right)=O\left(1\right)$$

*compared to O(N) for generative decoding, where N=dim(A). The spatial complexity reduction factor is:*

(45) $$\frac{\mathrm{params}(\mathrm{Decoder})}{\mathrm{params}(\mathrm{FiLM})}=\Omega \left(\frac{\dim(A)}{d_{z}\cdot C}\right)$$

*which is $∼10^{5}$ for typical medical volumes.*

**Proof.**  ${\mathrm{MLP}}_{\mathrm{FiLM}}$ maps $R^{2d_{z}}\to R^{2C}$ with hidden dimension $d_{h}$, requiring $\left(2d_{z}\cdot d_{h}+d_{h}\cdot 2C\right)$ parameters. With $d_{z}=128$, $d_{h}=64$, C=128: $\mathrm{params}\approx 128\times 64+64\times 256\approx 2.5\times 10^{4}=O\left(1\right)$. A generative decoder requires Ω(dim(A)) parameters to reconstruct the amplitude volume.    □

## 8. Phase Consistency Constraint

**Definition 9**  (Phase Consistency Loss)**.** *For S subbands total, the phase consistency loss measures cosine similarity between original and post-augmentation DTCWT phases:*

(46) $$L_{\mathrm{phase}}=1-\frac{1}{S}\sum_{s=1}^{S}\cos\left(\Phi_{s}^{\mathrm{orig}},\Phi_{s}^{\mathrm{aug}}\right)$$

*where $\cos\left(\theta_{1},\theta_{2}\right)=\frac{1}{|\theta_{1}|}\sum_{i}\cos\left(\theta_{1,i}-\theta_{2,i}\right)$ is the mean cosine of angular differences.*

**Proposition 8**  (Phase Consistency as Structural Fidelity)**.** *$L_{\mathrm{phase}}\to 0$ implies that the post-augmentation reconstruction preserves the DTCWT phase structure:*

(47) $$∥\Phi^{\mathrm{orig}}-\Phi^{\mathrm{aug}}{∥}_{\mathrm{circ}}\to 0$$

*where ${∥\cdot ∥}_{\mathrm{circ}}$ denotes the circular distance on [−π,π).*

**Remark 3**  (Domain-Dependence of $L_{\mathrm{phase}}$). *Unlike Sobel/SSIM-based structure preservation operating in the spatial domain, $L_{\mathrm{phase}}$ operates within the DTCWT domain, forming a closed optimization loop with the DTCWT front-end. This design ensures that structure preservation is inherently aligned with the operating space of the information bottleneck.*

## 9. Total Training Objective

Combining all components, the total training objective is

(48) $$L_{\mathrm{total}}=L_{\mathrm{seg}}+\lambda_{\mathrm{dis}}\left(L_{\mathrm{dom}}+\beta L_{\mathrm{KL}}\right)+\alpha L_{\mathrm{orth}}+\gamma L_{\mathrm{phase}}$$

**Definition 10**  (Loss Grouping and Hyperparameter Semantics)**.** *The loss terms are logically grouped into four categories:*

*(i)*  *Segmentation Bundle (weight 1.0, fixed): $L_{\mathrm{seg}}=L_{\mathrm{seg}}^{\mathrm{Dice}}+L_{\mathrm{seg}}^{\mathrm{CE}}$;*
*(ii)*  *Disentanglement Bundle (weight $\lambda_{\mathrm{dis}}$): $L_{\mathrm{dom}}+\beta L_{\mathrm{KL}}$, where β is the annealing coefficient (Equation (39)), not a hyperparameter;*
*(iii)*  *Orthogonality Term (weight α): $L_{\mathrm{orth}}$ as defined in Equation (36);*
*(iv)*  *Phase Term (weight γ): $L_{\mathrm{phase}}$ as defined above.*

**Remark 4**  (Effective Hyperparameter Count)**.** *The objective in Equation (48) has three tuneable weights ($\lambda_{\mathrm{dis}},\alpha ,\gamma$), not five, as it may appear. β is the annealing schedule coefficient, and the segmentation weight is fixed at 1.0. This logical grouping reduces the perceived hyperparameter space from $R_{+}^{5}$ to $R_{+}^{3}$.*

## 10. Two-Pass Test-Time Adaptation Theory

### 10.1. Uncertainty as Domain Shift Signal

Let $u\left(x\right)\in {\left[0,1\right]}^{D\times H\times W}$ be the model’s predictive uncertainty map (e.g., entropy of softmax outputs). Under domain shift, the model exhibits elevated uncertainty in regions where domain-specific amplitude characteristics mislead the segmentation head. Calibration is evaluated using the standard expected calibration error formulation [34].

**Assumption 1**  (Calibrated Uncertainty Transfer)**.** *The uncertainty head is ϵ-calibrated in the sense of Expected Calibration Error (ECE):*

(49) $$\mathrm{ECE}=E_{\hat{p}}\left[|P(\hat{y}=y|\hat{p}=p)-p|\right]\leq \epsilon$$

*where $\hat{p}$ is the model’s predicted confidence.*

### 10.2. Two-Pass Inference Algorithm

The inference procedure first identifies spatial regions with elevated predictive uncertainty, applies a localized correction only to the corresponding DTCWT amplitude coefficients, and then reconstructs the image while preserving its original phase. Algorithm 1 summarizes the complete two-pass procedure used in the subsequent analysis.

**Theorem 9**  (Two-Pass Generalization Gap Reduction)**.** *Under Assumption 1, the two-pass procedure described in Algorithm 1 achieves*

(50) $$R_{T}\left(\text{Two-Pass}\right)\leq R_{T}\left(\text{Single-Pass}\right)-\Delta_{\mathrm{adapt}}$$

*where $\Delta_{\mathrm{adapt}}=E_{x∼P_{T}}\left[1\left[u_{1}\left(x\right)>\tau \right]\cdot \delta_{\mathrm{local}}\left(x\right)\right]\geq 0$, with τ being a confidence threshold and $\delta_{\mathrm{local}}$ the expected local improvement from amplitude correction in high-uncertainty regions.*

**Proof Sketch.**  The proof decomposes the domain generalization error into calibrated and uncalibrated components. Under ECE ≤ϵ, the uncertainty $u_{1}$ serves as a noisy but unbiased signal of domain-induced errors. The soft mask m localizes corrections to high-uncertainty regions, avoiding unnecessary perturbation of well-generalized regions. The DTCWT amplitude correction $\tilde{A}$ reduces the local amplitude discrepancy $\left|\tilde{A}-A_{T}\right|<\left|A_{1}-A_{T}\right|$, where $A_{T}$ is the amplitude of a hypothetical in-domain image. Since the model’s error increases monotonically with amplitude discrepancy (by the Lipschitz property of the segmentation head), the corrected input yields lower expected error.    □

**Algorithm 1:** Two-Pass Uncertainty-Guided DTCWT Adaptation
Given a trained segmentation model $f_{\theta }$, an uncertainty head $u_{\theta }$, and a test image x:(1)First pass: compute the initial segmentation logits and uncertainty map,$$\left({\hat{y}}_{1},u_{1}\right)=f_{\theta }\left(x\right),u_{\theta }\left(x\right).$$(2)Wavelet decomposition: compute DTCWT coefficients$$W\left(x\right)=A_{1}⊙e^{i\Phi_{1}}.$$(3)Mask construction: convert the uncertainty map into a smooth spatial mask$$m=\mathrm{Smooth}\left(1[u_{1}>\tau ]\right),$$
where τ is an uncertainty threshold.(4)Amplitude correction: apply local amplitude normalization or modulation only inside high-uncertainty regions:$\tilde{A}=\left(1-m\right)⊙A_{1}+m⊙\mathrm{Norm}\left(A_{1}\right).$(5)Reconstruction: reconstruct the adapted image while preserving the original phase:$\tilde{x}=W^{-1}\left(\tilde{A}⊙e^{i\Phi_{1}}\right).$(6)Second pass: compute the final prediction${\hat{y}}_{2}=f_{\theta }\left(\tilde{x}\right).$
The output of the procedure is ${\hat{y}}_{2}$.

**Theorem 10**  (Two-Pass Complexity)**.** *The two-pass procedure requires exactly two forward passes and one DTCWT inverse transform. Compared to Monte Carlo dropout with N samples:*

(51) $$\frac{\mathrm{cost}(\text{Two-Pass})}{\mathrm{cost}(\mathrm{MC}\;{\mathrm{Dropout}}_{N})}=\frac{2}{N}\overset{N\to \infty }{\to }0$$

**Proof.**  Each DTCWT inverse requires O(D·H·W·log(HW)) operations—one FFT per slice. For 240×240×155 volumes, the cost ratio is ≈2/N. With typical N=30 for MC dropout, the two-pass procedure is 15× faster. □

## 11. Discussion

### 11.1. Physical Priors as Dimensionality Relief

Our framework illustrates a general principle: physical priors can reduce the effective dimensionality of representation learning. The DTCWT’s structured decomposition effectively sparsifies the information bottleneck problem by pre-separating structure (phase) from style (amplitude). This reduces the effective dimensionality from $O(10^{5})$ (raw spatial domain) to $O(10^{2})$ (compressed amplitude statistics), enabling the VIB to operate efficiently.

We conjecture that this principle extends beyond medical imaging to any domain where a physically motivated signal decomposition reveals a natural separation of task-relevant and domain-specific information.

### 11.2. Connections to Domain Generalization Theory

Our work connects to several lines of theoretical DG research:

(i) Ben-David et al. bound [1]: Under the phase–amplitude premise, the DTCWT front-end can be viewed as a representation mapping *g* intended to reduce acquisition-driven components of the HΔH-divergence between source and target domains.
(ii) Invariant Risk Minimization (IRM) [19]: The triple-constraint mechanism implements a structured invariance objective: task features are encouraged to be invariant across domains, while domain features capture controlled variation.
(iii) Domain-Adversarial Training [20]: Domain supervision provides an alternative to adversarial training, avoiding the min–max instability while explicitly retaining a compressed domain code.

### 11.3. Verifiability

A defining feature of our theoretical framework is its verifiability through clear experimental predictions:

(1) Amplitude-phase swap: If Property 2 is not useful in a given dataset, then amplitude swapping between domains should not yield the predicted acquisition-style perturbations and may substantially degrade segmentation.
(2) Ablation non-redundancy: If Theorem 6 is reflected in a practical implementation, then progressive ablation is expected to show: (B) < (A), (C) > (B), (D) > (C), (E) > (D)—where violations would indicate that the corresponding constraint is not contributing as predicted.
(3) VIB compression: If the VIB is effective, the domain classifier accuracy should remain high while KL compression increases over training, indicating that domain information is being compressed rather than discarded.

### 11.4. Validation Logic

The two empirical checks below are designed to test different parts of the theoretical argument rather than to serve as a complete benchmark. The FeTS site-classification experiment tests the premise that DTCWT amplitude carries more domain/site information than phase. The BraTS segmentation experiment tests a weaker but necessary implementation condition: a DTCWT front end must remain compatible with a real public medical segmentation task and must not collapse segmentation performance. Table 4 summarizes how each check maps to the corresponding theoretical claim.

### 11.5. Illustrative Empirical Sanity Check

Although the main contribution of this paper is theoretical, we performed a lightweight sanity validation of the phase–amplitude premise using the FeTS 2022 multi-site brain tumor segmentation data [35]. The purpose is not to provide a full segmentation benchmark, but to test whether DTCWT amplitude statistics carry more site/domain information than DTCWT phase statistics. This directly addresses the weakest point of the theory: if amplitude and phase were equally site-discriminative, then the proposed phase–amplitude separation would have little empirical support.

We selected five FeTS sites with sufficient sample counts, sampled eight cases per site, and extracted five non-empty axial slices per case. For each case, we computed a two-level axial DTCWT and summarized amplitude and phase subbands using simple per-channel and per-orientation statistics. A balanced logistic regression classifier was then trained to predict site identity using stratified five-fold cross-validation. Table 5 reports the resulting domain classification performance.

These results support the structural premise that site/domain information is more concentrated in DTCWT amplitude features than in phase features. They should be interpreted conservatively: the experiment validates the plausibility of the representation assumption under the sampled FeTS setting, but it does not replace a full segmentation benchmark or prove that the premise holds under all medical imaging shifts. The result is nevertheless informative because it tests the premise using only low-capacity summary features and a simple linear classifier; the observed gap is therefore not a consequence of a large segmentation network learning arbitrary site cues.

### 11.6. Illustrative Public-Dataset Segmentation Check

We also evaluated whether the DTCWT front-end can be instantiated in a public medical image segmentation setting. Using the public BraTS 2023 adult glioma challenge data [36], we trained DTCWT-UNet variants with one, two, and three DTCWT decomposition levels and evaluated them on the held-out internal validation split used by our implementation. All three completed runs used the same backbone width, training schedule, and parameter budget (17.5 M trainable parameters; 150 epochs; batch size 2). The evaluation included 251 validation cases and reports per-case Dice scores for the BraTS tumor regions: whole tumor (WT), tumor core (TC), and enhancing tumor (ET). This experiment addresses a different concern from the FeTS site-classification test: it checks that the DTCWT representation can be used in an actual public segmentation workflow, rather than only in an abstract site-classification analysis. The results of the three completed DTCWT-UNet configurations are reported in Table 6.

This segmentation check is deliberately limited: it verifies that DTCWT-based representations can support non-trivial public brain-tumor segmentation performance, but it is not presented as a controlled state-of-the-art comparison. Only completed and internally consistent DTCWT-front-end runs are reported. Together with Table 5, the results provide empirical support for the central representation premise while preserving the paper’s main scope as a conditional mathematical analysis.

### 11.7. Limitations

We note several theoretical limitations:

(i) Axial-plane approximation: The 2D axial DTCWT provides an upper bound on through-plane information loss, but the bound’s tightness depends on the acquisition parameters.
(ii) ECE assumption: Theorem 9 depends on calibrated uncertainty, which may not hold under extreme domain shifts.
(iii) PAC-Bayesian bounds: The finite-sample bounds (Theorem 2) involve complexity terms that are loose for deep networks, suggesting room for tighter analysis using modern generalization theory.
(iv) Empirical scope: The FeTS and BraTS checks are illustrative validations of the representation premise and implementation feasibility. They do not constitute a full multi-dataset benchmark, do not establish state-of-the-art segmentation performance, and do not test every component of the full training objective.
(v) Domain-shift scope: The phase–amplitude premise is most appropriate for acquisition-driven appearance shifts after spatial normalization. It may be weaker for cross-modality shifts, severe pathology-induced deformation, missing sequences, or shifts where anatomy and acquisition style are entangled.
(vi) Independence claims: The orthogonality constraint controls linear cross-subspace leakage in finite batches. Statistical independence or mutual-information separation requires additional distributional and sufficiency assumptions; the revised theorems state this explicitly.

## 12. Conclusions

We have presented a mathematical framework for domain generalization motivated by complex wavelet decomposition and variational information theory. The key theoretical contributions include: (1) formalization of the local phase-magnitude complementarity premise that motivates DTCWT as a front-end for domain analysis; (2) a conditional analysis showing when amplitude subbands concentrate domain-relevant information more effectively than global Fourier representations; (3) a triple-constraint non-redundancy analysis showing that domain supervision, KL compression, and orthogonality address distinct failure modes; (4) a predictive modulation scheme achieving O(1) spatial parameter complexity with respect to the amplitude volume while preserving information bottleneck semantics; and (5) a two-pass adaptation theory with a sufficient condition for generalization gap reduction under calibration assumptions.

The framework is designed to be verifiable: its main assumptions and conditional results map to concrete experimental predictions. The illustrative FeTS and BraTS checks do not convert the paper into a complete empirical benchmark, but they test whether the central phase–amplitude premise is plausible and whether a DTCWT front end can support public medical segmentation. While developed in the context of medical image segmentation, the principles of physically motivated information bottleneck disentanglement may extend to other domains where signal structure can reduce the dimensionality of representation learning.

## Figures and Tables

**Table 1:** Relationship between established formulations and the proposed theory.

Component	Status	Role in This Paper
Ben-David style DG bounds	Prior work	Motivation for reducing domain divergence
IB/VIB objective	Prior work	Compression language for latent domain codes
Domain cross-entropy loss	Prior work	Supervised identifiability signal for $Z_{\mathrm{domain}}$
Gaussian KL term	Prior work	Computable capacity control for $Z_{\mathrm{domain}}$
FiLM modulation	Prior work	Lightweight conditional modulation operator
DTCWT subband bottleneck	Proposed	Applies VIB to localized amplitude representations
Triple-constraint analysis	Proposed	Identifies distinct failure modes addressed by $L_{\mathrm{dom}}$, $L_{\mathrm{KL}}$, and $L_{\mathrm{orth}}$
Two-pass DTCWT adaptation bound	Proposed	Provides a sufficient condition for uncertainty-guided amplitude correction

**Table 2:** Systematic comparison with representative domain generalization and segmentation frameworks.

Framework	Mechanism and Controlled Quantity	Relation to This Work
Global Fourier DG [3,4]	Global amplitude manipulation controls image-level style.	Motivates frequency-domain DG, but lacks spatial locality; DTCWT uses localized complex subbands.
Adversarial DG [20,21]	Min–max domain confusion controls domain predictability.	Suppresses domain information; our framework retains a compressed domain code $Z_{\mathrm{domain}}$ for modulation.
IRM/invariant features [18,19]	Stable predictors control environment-dependent risk.	Does not specify a physical signal decomposition; our premise is tied to medical phase–amplitude structure.
IIB [16]	Mutual-information regularization controls invariant capacity.	Shares the IB language, but applies it to generic features rather than DTCWT amplitude subbands.
mDSDI/INSURE [17,23]	Disentanglement controls invariant/domain-specific factors.	Closest in representation objective; our analysis is conditional on a physically motivated wavelet domain.
SAM/MedSAM [27,28]	Pretraining and prompting control cross-task transfer.	Addresses another generalization axis; our theory targets site/acquisition shifts in a fixed task.
Proposed framework	DTCWT bottleneck controls local domain information, leakage, and calibrated adaptation.	Provides conditional bounds and failure-mode analysis for a signal-structured domain representation.

**Table 3:** Originality boundary and assumptions of the main theoretical results.

Result	Inherited Component	Proposed Step	Main Assumption or Limitation
Property 2	Classical phase importance and MRI acquisition physics	Formulates a local phase–magnitude complementarity premise for DG analysis	Structural premise; not a universal law and must be empirically checked.
Theorem 1	Data processing and wavelet locality arguments	Compares local DTCWT amplitude information with global FFT-style representations	Requires localized domain shifts and regularity of the domain transform.
Theorem 2	IB/VIB and PAC-Bayesian upper-bound machinery	Applies capacity control to a DTCWT amplitude-domain code $Z_{\mathrm{domain}}$	Bound is loose for deep networks and functions as a capacity-control rationale.
Theorem 5	Linear covariance control	Replaces the stronger independence claim with linear cross-subspace leakage control	Independence follows only under additional linear-Gaussian and sufficiency assumptions.
Theorem 6	Standard domain loss, KL loss, and orthogonality regularization	Identifies the distinct failure mode removed by each constraint	Conditional non-redundancy, not an unconditional disentanglement guarantee.
Theorem 9	Calibration-based uncertainty reasoning	Derives a sufficient condition for uncertainty-guided DTCWT amplitude correction	Depends on calibrated uncertainty transfer under target-domain shift.

**Table 4:** How the illustrative empirical checks map to the theoretical claims.

Theoretical Issue	Empirical Check	Positive Evidence Expected	Remaining Limitation
Amplitude carries site information	FeTS site classification from DTCWT summaries	Amplitude features predict site better than phase features and above chance	Does not prove the premise for every scanner, anatomy, or modality
Phase is less domain-specific	Same FeTS comparison using phase features	Phase features show weaker site prediction than amplitude features	Phase can still carry domain information under misregistration or structural shift
DTCWT front end is usable for segmentation	BraTS DTCWT-UNet validation	Non-trivial Dice on public brain-tumor segmentation cases	Not a controlled SOTA comparison and not a full ablation of all losses
Theoretical claims are conditional	Conservative interpretation of both checks	Results support plausibility rather than universal validity	Broader validation across centers and modalities remains necessary

**Table 5:** Sanity validation of the DTCWT phase–amplitude premise on FeTS 2022 site classification. Chance accuracy is 0.20 because five sites are used. Values are mean ± standard deviation over stratified five-fold cross-validation.

Feature Set	Accuracy	Macro-F1
DTCWT amplitude statistics	0.700±0.127	0.668±0.184
DTCWT phase statistics	0.450±0.127	0.405±0.092
Amplitude + phase statistics	0.775±0.184	0.695±0.247

**Table 6:** Illustrative BraTS 2023 segmentation check for completed DTCWT-front-end configurations. Values are per-case Dice on 251 validation cases. Mean Dice is reported as mean ± standard deviation; WT, TC, and ET columns report class-wise means.

Config.	Cases	Mean Dice	WT	TC	ET
DTCWT-UNet, J=1	251	0.717±0.172	0.649	0.750	0.752
DTCWT-UNet, J=2	251	0.731±0.157	0.658	0.782	0.752
DTCWT-UNet, J=3	251	0.725±0.163	0.654	0.770	0.751

## Data Availability

No new datasets were created in this study. The illustrative sanity validation used the publicly available FeTS 2022 data, and the segmentation feasibility check used the public BraTS 2023 challenge data. The code, case lists, experiment provenance notes, and derived summary statistics supporting the illustrative validation checks are available at https://github.com/frankliuf/entropy-dtcwt-revision-artifacts (accessed on 28 June 2026). Raw FeTS and BraTS medical images are not redistributed in that repository and should be obtained from their official data-access portals under the corresponding data-use agreements.

## Abbreviations

The following abbreviations are used in this manuscript:

DTCWT	Dual-Tree Complex Wavelet Transform
VIB	Variational Information Bottleneck
DG	Domain Generalization
KL	Kullback–Leibler
IB	Information Bottleneck
FiLM	Feature-Wise Linear Modulation
ECE	Expected Calibration Error
FFT	Fast Fourier Transform
IRM	Invariant Risk Minimization
MRI	Magnetic Resonance Imaging
